# A Dual Branch Fusion Network for Simultaneous Tea Leaf Disease Diagnosis and Age-Based Quality Grade Evaluation

**DOI:** 10.3390/plants15152391

**Published:** 2026-08-04

**Authors:** Xin Zhang, Jiahua Ren, Siyu Qin, Xiu Zhang

**Affiliations:** 1 Interdisciplinary Center, Tianjin Normal University, Tianjin 300387, China; ecemark@tjnu.edu.cn (X.Z.);; 2 Tianjin Key Laboratory of Wireless Mobile Communications and Power Transmission, Tianjin Normal University, Tianjin 300387, China; 3 College of Artificial Intelligence, Tianjin Normal University, Tianjin 300387, China

**Keywords:** precision agriculture, deep learning, multi-task learning, tea leaf disease and pest-damage, tea leaf-age quality grading

## Abstract

The simultaneous diagnosis of diseases and evaluation of age quality grades in tea leaves are critical for precision agriculture and the economic valuation of tea products. Although deep learning has shown promise in agricultural vision tasks, current multi-task models often suffer from performance degradation due to feature conflicts: tea leaf disease recognition relies heavily on macro-structural lesions, whereas tea leaf-age quality grading depends on micro-textural features such as trichome density and color uniformity. To address this discrepancy, we propose a novel dual branch fusion network. Our architecture fundamentally decouples the feature extraction process by utilizing a dual branch mechanism. The first branch employs global average pooling to capture first-order spatial statistics; it can retain the global structural layout necessary for macro-lesion detection. The second branch introduces a dimensionality-reduced self-bilinear pooling module to compute second-order covariance matrices; it can effectively capture the fine-grained textural patterns essential for micro-grade classification. These decoupled features are subsequently fused and optimized through a weighted multi-task loss function. Experimental results on a comprehensive tea leaf dataset demonstrate that the proposed dual fusion framework significantly outperforms baseline models. The proposed network can rescue the disease classification accuracy drop observed in standard bilinear models while maintaining exceptional grading performance. Furthermore, the proposed network maintains a compact parameter footprint and low computational complexity. This balance renders it suitable for deployment on agricultural Internet of Things edge devices where inference speed is critical.

## 1. Introduction

Tea stands as one of the most widely consumed non-alcoholic beverages globally, underpinning a massive agricultural industry that supports the livelihoods of millions of farmers [1]. The tea industry is a cornerstone of the agricultural economy in many developing nations, particularly in Asia and Africa. The economic viability of tea production hinges critically on two important factors: the health status of the tea plants and the quality grade of the harvested leaves [2]. On one hand, various foliar diseases can cause devastating yield losses, reducing photosynthetic efficiency and rendering leaves unmarketable. On the other hand, the market value of tea is strictly stratified by plucking standards, typically categorized into grades ranging from single bud to one bud with three or more leaves. Premium grades command exponentially higher prices due to their superior flavor profiles and chemical compositions. Consequently, the simultaneous and accurate assessment of disease presence and leaf-age quality grade is not merely an agronomic necessity but a fundamental economic imperative for modern precision tea management [3,4].

Traditionally, the diagnosis of tea leaf diseases and tea leaf quality grading have relied heavily on manual inspection by experienced agronomists. Although this approach leverages human expertise, it is inherently subjective, labor-intensive, time-consuming, and prone to fatigue-induced errors, especially when deployed across vast plantation areas [5,6]. In recent years, the advent of deep learning (DL) and computer vision (CV) has revolutionized agricultural phenotyping, offering automated solutions for plant disease detection [7] and crop classification [8]. Convolutional Neural Networks (CNNs), including architectures like ResNet, ShuffleNet, and MobileNet, have demonstrated remarkable success in identifying obvious visual symptoms of plant stress. However, despite these advancements, the specific domain of tea leaf analysis presents a unique set of challenges that remain largely unresolved by current state-of-the-art (SOTA) methodologies. These challenges stem from the intrinsic visual characteristics of tea leaves and the structural limitations of existing computational frameworks [9].

Furthermore, the prevailing paradigm in agricultural artificial intelligence (AI) research treats disease detection and quality grading as disjoint, single-task problems. Existing literature typically develops separate models for each objective: one network trained to classify diseases [10] and another independent network trained to predict leaf grades [11]. This siloed approach introduces significant inefficiencies. From a computational perspective, deploying multiple heavy-weight models on edge devices such as handheld scanners or agricultural drones exacerbates latency and energy consumption, hindering real-time application in resource-constrained field environments [12]. More critically from a biological perspective, this separation ignores the inherent synergistic relationship between plant health and developmental stage [13]. Agronomic knowledge suggests that susceptibility to specific pathogens is often correlated with leaf maturity; for instance, high-grade leaves like tender buds may be more prone to certain fungal infections like Blister Blight compared to mature, coarse leaves [14]. By training models in isolation, we fail to leverage this latent correlation, missing an opportunity for the model to learn shared representations that could enhance the generalization capability of both tasks simultaneously. The lack of a unified framework that can jointly reason about health and quality represents a significant gap in the current landscape of smart tea agriculture.

To address these compounded challenges, there is an urgent need for a novel DL architecture that can explicitly model fine-grained textural features while efficiently handling multiple correlated tasks within a unified inference pipeline. The core of this solution lies in moving beyond first-order feature aggregation. Bilinear Pooling, a technique originally developed for fine-grained visual categorization in general CV, offers a promising mathematical foundation. By computing the outer product of feature vectors, bilinear pooling captures pairwise interactions between feature channels, effectively encoding second-order statistics that represent texture and local structure. However, the direct application of standard bilinear models is often prohibitive due to the explosive dimensionality of the resulting feature vectors, leading to excessive parameter counts and computational overhead. Moreover, integrating this mechanism into an efficient, multi-task framework specifically tailored for the complex background of tea plantations remains an unexplored frontier.

In this study, we propose GBDFNet, a novel global-bilinear dual fusion network designed specifically for the simultaneous diagnosis of tea leaf diseases and assessment of leaf-age quality grades. Our approach fundamentally rethinks the feature extraction process by introducing a dual-stream architecture that synergistically combines macroscopic semantic cues with microscopic textural details. The first stream employs global average pooling to retain first-order global features essential for identifying broad disease categories and leaf shapes. The second stream introduces a self-bilinear pooling module, which explicitly models channel-wise correlations to capture the subtle texture variations critical for distinguishing early-stage infections and fine-grained quality levels. To mitigate the computational burden associated with bilinear features, we incorporate a dimensionality reduction strategy that compresses the high-order feature space without sacrificing discriminative power. These two complementary feature streams are then fused and fed into a multi-task head, enabling the simultaneous prediction of two key attributes: specific disease and pest-damage type and leaf-age quality grade.

The core innovation of this paper lies in how we handle the conflicting feature requirements of disease and quality tasks. Most existing models treat these as separate problems or use a single feature extractor for both. We argue that this is insufficient. Tea leaf disease recognition needs to see the big picture like lesion shapes. Tea leaf quality grading relies on tiny details like hair density and texture. Our method solves this by decoupling the feature extraction process. We explicitly introduce self-bilinear pooling to tea analysis for the first time. This allows the network to mathematically model second order statistics which represent texture. Standard CNNs often miss these subtle cues. By fusing this texture specific branch with a global semantic branch, we create a model that understands both the forest and the trees. This design not only boosts accuracy but keeps the model efficient enough for edge devices. Specifically, the innovations are:

(1) Introduction of self-bilinear pooling to tea analysis: To the best of our knowledge, this is the first study to explicitly incorporate self-bilinear pooling mechanisms into the domain of tea leaf analysis. By mathematically modeling the second-order statistics of feature maps, GBDFNet overcomes the texture-blindness of traditional CNNs, significantly enhancing the model’s sensitivity to fine-grained visual cues such as early fungal sporulation and subtle vein patterns associated with leaf maturity.
(2) Design of a dual branch feature fusion architecture: We propose a novel architectural design that harmonizes global semantic information and local textural correlations. This hybrid approach ensures that the model possesses a comprehensive visual understanding, capable of recognizing both the overall leaf health and shape and the microscopic disease spots and grade-specific textures, thereby addressing the core challenge of visual similarity in fine-grained tea classification.
(3) Development of an efficient multi-task learning framework: Breaking away from the conventional single-task paradigm, we construct a unified multi-task learning head that jointly optimizes for disease and pest-damage classification and leaf-age quality grading. This design not only reduces the computational footprint and inference latency, but also leverages the biological interdependencies between tasks to improve overall model performance through shared representation learning.

By bridging the gap between advanced second-order feature modeling and practical agricultural needs, this paper offers a robust, scalable, and scientifically grounded solution for the tea industry. It paves the way for the next generation of intelligent agricultural systems capable of making nuanced, multi-faceted decisions in real-time, ultimately contributing to sustainable tea production and enhanced economic returns for growers.

The rest of the paper is organized as follows: a brief overview of the current state of research on tea leaf disease and pest-damage recognition and leaf-age quality grading is given in Section 2. The proposed method in this paper is described in detail in Section 5. Section 3 presents the experiments and results. Section 4 discusses the biological interpretation, deployment feasibility, and limitations of the proposed method. Section 6 concludes the paper.

## 2. Background

In this section, we review related works on tea leaf disease recognition and tea leaf-age quality grading.

### 2.1. Deep Learning in Tea Leaf Disease Recognition

Automated tea leaf disease recognition is a multi-faceted challenge. To enhance model robustness in complex field conditions, Li and Liao [15] integrated two-stage image segmentation with IC-GAN for tea leaf data augmentation and disease recognition, whereas Ye et al. [16] utilized EnlightenGAN with YOLOv8 to address low-light detection. The evolution of detection architectures has been summarized in a comprehensive review of the YOLO family for plant diseases [17], and specific lightweight models like EfficientNetB0 with efficient channel attention [18] have been proposed for mobile deployment. Furthermore, MAF-MixNet [19] was developed to tackle few-shot learning for rare diseases. Beyond recognition, recent biological research has explored the impact of specific pathogens, such as Sooty Mould on photosynthesis [10] and the application of fern-based biopesticides against Red Spider Mites [20]. Although these methods excel in specific tasks, they often rely on standard first-order convolutions which may fail to capture the high-order textural nuances essential for distinguishing morphologically similar diseases.

### 2.2. Deep Learning in Tea Leaf-Age Quality Grading

The quality of tea leaf is determined by a complex interplay of morphology, chemistry, and environment [21,22]. Vision-based quality grading has advanced through models like YOLOv8x-SPPCSPC-CBAM for bud-leaf classification [23] and YOLOv11-PFT for non-destructive micro-impurity detection [24]. Other researchers have leveraged Fourier transform near infrared spectroscopy combined with CNNs to predict polyphenol content [25] or used hyperspectral imaging to grade red scab severity [26]. Chemical studies provide the theoretical basis for these visual cues: Zheng et al. [27] and De et al. [28] identified seasonal and grade-based metabolic variations in Oolong and Darjeeling teas, whereas Ye et al. analyzed the sensory quality of different tea powders [29]. Additionally, environmental factors such as extreme temperatures [30] and ozone levels [11] have been shown to significantly affect tea plant resistance and leaf quality. However, most existing vision models process tea leaves either as a whole or as localized patches, failing to effectively decouple and fuse the macro-structure and micro-texture information.

### 2.3. Advancements in Deep Learning for Fine-Grained Tasks

In the broader context of agricultural deep learning, the focus is shifting toward capturing more discriminative features. Current literature frequently cites the trade-off between speed and accuracy, as seen in various YOLO-based applications [31] and lightweight CNNs [18,32]. Despite these advancements, standard architectures often lack the mechanism to model second-order statistics, which are vital for fine-grained tasks. Our proposed GBDFNet bridges this gap by introducing a dual-path architecture. First-order global features can capture the macro-morphology discussed in grading studies; whereas second-order bilinear features can capture the micro-textural patterns relevant to early disease symptoms. By fusing first-order global features with second-order bilinear features, GBDFNet provides a more holistic and precise representation of tea leaf conditions than the single-path models prevalent in current literature.

## 3. Experimental Results

In this section, we will conduct experiments on TeaLeafDiseaseQuality dataset to validate the performance of the proposed GBDFNet method.

### 3.1. Implementation Details

All experiments are conducted on a high-performance workstation equipped with an NVIDIA GeForce RTX 3090 GPU and an Intel Core i7 processor, running on the Windows 11 operating system. The proposed GBDFNet method is implemented using the PyTorch deep learning library. The source code and trained models will be released publicly upon acceptance. We used Python 3.9.18, PyTorch 2.1.0, torchvision 0.16.0, and other standard scientific libraries. Random seeds were fixed to 42. For CUDA operations, we set torch.backends.cudnn.deterministic to True and torch.backends.cudnn.benchmark to False to ensure reproducible results. All pretrained weights were loaded directly from torchvision’s official model weights. Specifically, we used MobileNet_V3_Small_Weights.IMAGENET1K_V1, ShuffleNet_V2_X1_0_Weights.IMAGE NET1K_V1, MNASNet1_0_Weights.IMAGENET1K_V1, and yolo11n-cls. These weights are pretrained on the ImageNet-1K dataset and are publicly available through the PyTorch model zoo.

The input images are resized to 224×224 pixels. The hyperparameters are given in Table 1. We employ the AdamW optimizer, setting the initial learning rate to $1\times 10^{-3}$ with a weight decay of $1\times 10^{-5}$ to prevent overfitting. A cosine annealing learning rate scheduler is utilized to dynamically adjust the learning rate over the course of training, allowing the model to converge smoothly into sharp minima. The total number of training epochs is set to 50, with an early stopping mechanism triggered if the validation loss did not improve for 10 consecutive epochs. The batch size was fixed at 32 to balance memory consumption and gradient stability.

### 3.2. Evaluation Metrics

Model performance is evaluated using standard classification metrics: accuracy (ACC), F1-score (F1), and mean average precision (mAP). To ensure statistical reliability, we conduct six independent experiments using stratified five-fold cross-validation as described in Section 5.1.1. The final performance metric of the model is obtained by averaging the evaluation results. The inference speed is measured in floating-point operations per second (FLOPS) on the target GPU to assess real-time deployment feasibility.

### 3.3. Quantitative Multi-Task Performance

To comprehensively evaluate the efficacy and robustness of our proposed GBDFNet, we conduct extensive comparative experiments against four representative baseline models spanning different architectural paradigms and eras of development. The selection of these baselines is driven by three key criteria: architectural diversity, computational efficiency relevance, and state-of-the-art timeliness.

For lightweight mobile architectures, we select MobileNetV3-Small [33], ShuffleNet [34], and MnasNet [35] as primary baselines because they represent the gold standard for edge-computing scenarios. Given that our target application involves deploying disease detection models on handheld devices or drones in tea gardens, comparing against these highly optimized networks is crucial. MobileNetV3-Small utilizes neural architecture search (NAS) to optimize latency-accuracy trade-offs. ShuffleNet introduces channel shuffling to mitigate the information loss caused by group convolutions. MnasNet explicitly incorporates hardware latency into its reward function during the search process. Comparing GBDFNet against these models validates whether our method offers a superior balance between inference speed and accuracy in resource-constrained environments.

For real-time detector, we select YOLOv11n-cls as baseline. To ensure the timeliness and competitiveness of our evaluation, we included YOLOv11n-cls [24], the latest iteration in YOLO series. YOLOv11 represents the current good model in real-time object detection and classification, incorporating advanced attention mechanisms and optimized backbone structures. By comparing GBDFNet with YOLOv11n-cls, we rigorously test our method against the most modern and powerful architectures available today. This comparison demonstrates whether our proposed multi-task fusion strategy can compete with or exceed the performance of cutting-edge, large-scale detection frameworks, even with a significantly lighter parameter footprint.

In summary, this diverse set of baselines covers the spectrum from efficient NAS-derived models to the latest real-time giants. This comprehensive selection ensures that the performance gains reported for GBDFNet are robust under similar acquisition conditions.

Table 2 presents a comparative analysis of computational complexity and inference efficiency across different model architectures. Although ShuffleNet exhibits the lowest parameter count (i.e., 1.266 M) and MobileNetV3-Small demonstrates the lowest floating-point operations (i.e., 60.87 M), these metrics do not fully translate to real-time performance on modern hardware.

It is important to note that FLOPS are a theoretical metric that does not always correlate perfectly with real-world inference latency. The significant speed advantage of GBDFNet can be attributed to its hardware-friendly design. Our architecture utilizes 1 × 1 convolutions and matrix operations in the bilinear branch which achieve higher arithmetic intensity and better GPU utilization compared to the depthwise separable convolutions found in MobileNetV3-Small. Furthermore, the parallel nature of our dual-stream processing reduces memory access overhead. This allows GBDFNet to maximize the throughput of the hardware accelerator resulting in the observed low latency.

Notably, our proposed GBDFNet achieves the fastest inference speed, recording a latency of only 0.29 ms on an NVIDIA RTX 3090 GPU. This represents a significant improvement, being approximately 2× faster than the nearest competitor, YOLOv11n-cls, and more than 2.3× faster than MobileNetV3-Small. Although GBDFNet has a slightly larger parameter size than the ultra-light baselines, its architectural design prioritizes computational efficiency. This approach effectively minimizes inference latency, proving that parameter count alone does not dictate deployment feasibility. These results confirm that GBDFNet strikes an optimal balance between model capacity and execution speed, demonstrating its strong potential for real-time tea leaf disease detection systems where low latency is critical.

Table 3 summarizes the performance comparison of different models on the tea leaf disease and pest-damage classification and detection task. Statistical tests are conducted on the results. Specifically, for each comparison, we performed paired *t*-test between the GBDFNet and each baseline model. For every pairwise comparison, we calculated the *p* value and the 95% confidence intervals (CI) of the mean performance differences. In Table 3, the symbol * means that the GBDFNet shows significant better performance than the associated baseline model. The CI is given in the last column of Table 3. The results demonstrate that the proposed GBDFNet significantly outperforms all baseline models on ACC metric.

Specifically, GBDFNet achieves an ACC of 93.12%, a macro F1-score of 92.51%, and an mAP of 98.04%. In contrast, the baseline models show inconsistent performance. Although MobileNetV3-Small achieves a competitive mAP of 94.57%, its accuracy 86.40% and F1-score 86.58% lag behind our method by approximately 6.72% and 5.93%, respectively. Other baselines, such as ShuffleNet and YOLOv11n-cls, exhibit substantial performance degradation, with ACC dropping below 65% and F1-scores falling under 65%, indicating their limited capacity to handle the complex spectral and spatial features of tea leaf diseases.

Furthermore, GBDFNet demonstrates superior stability, evidenced by the consistently low standard deviations (e.g., ±1.20% for mAP) compared to the high variance observed in ShuffleNet and YOLOv11n-cls. These results confirm that GBDFNet not only delivers better ACC result but also maintains performance capability across different data splits, making it highly reliable for practical agricultural applications.

Table 4 presents the performance comparison of different models on the tea leaf-age quality grading task. This task proves to be significantly more challenging than disease and pest-damage detection, as evidenced by the relatively high standard deviations across all models (e.g., ±13.69% for MobileNetV3-Small), likely attributable to the subtle visual distinctions between quality grades and inherent subjectivity in manual labeling. Paired *t*-test are also conducted on the results between GBDFNet and each baseline model. Symbol * indicates that GBDFNet significantly outperforms the associated baseline model with 0.05 significant level. The corresponding confidence intervals are also given in Table 4.

Despite these challenges, our proposed GBDFNet achieves the best overall performance, securing the highest ACC 85.58%, F1-score 86.66%, and mAP 95.99%. Although MnasNet demonstrates competitive stability with the lowest variance 8.11% in accuracy and a mean ACC of 66.17%, it still lags behind GBDFNet by approximately 19.41% in accuracy and 21.48% in F1-score. Other baselines, such as ShuffleNet and YOLOv11n-cls, struggle to generalize effectively, with accuracies dropping below 83% and exhibiting large performance fluctuations.

Notably, although GBDFNet exhibits a standard deviation of 11.49%, which is consistent with the high variance observed in this difficult task, its superior mean scores indicate a stronger capability to extract discriminative features for fine-grained quality classification. These results confirm that GBDFNet provides the most accurate solution for tea leaf-age quality grading, outperforming existing lightweight architectures even under conditions of high data variability.

**Figure 1:**
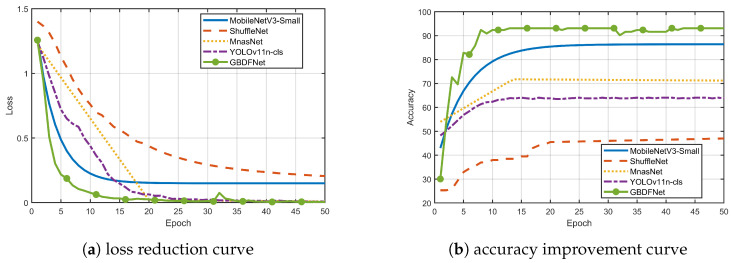
Learning curve analysis for all models. (**a**) loss reduction over training epochs. (**b**) accuracy progression over training epochs.

As shown in Figure 1, we evaluated the training efficiency of all five models. The loss curves in subfigure Figure 1a reveal that GBDFNet achieves rapid minimization of the objective function. It drops to near zero values significantly faster than MobileNetV3-Small or ShuffleNet. This indicates a more stable optimization landscape for our proposed method. Correspondingly, the accuracy trends in subfigure Figure 1b highlight the practical benefit of this convergence. GBDFNet surpasses the 90 percent threshold within just ten epochs. It maintains this superior performance without fluctuation. In comparison, standard baselines like MnasNet and YOLOv11n-cls plateau at lower accuracy levels around 64 to 71 percent. ShuffleNet shows the weakest performance, struggling to exceed 47 percent accuracy even after extensive training. These results collectively suggest that the architectural design of GBDFNet facilitates better feature extraction and gradient flow during the early stages of learning.

### 3.4. Ablation Study

To validate the effectiveness of the proposed GBDFNet, we conduct comprehensive ablation experiments. We systematically evaluate the contribution of each feature extraction branch: the GAP branch and the bilinear branch. The GAP branch captures global semantic context; whereas the the bilinear branch models second-order feature interactions for fine-grained texture analysis. Moreover, we have added a set of independent single-task experiments where the same backbone is trained separately for grade classification and disease and pest-damage classification using the same training protocol. The models are denoted as GAP Independent and Bilinear Independent, which are the single-task version of the GAP-only and bilinear-only, respectively. These independent models do not share any parameters or learning signals between tasks.

The experiments are performed under identical training conditions. When the bilinear branch is removed, the model is denoted by GAP Only. When the GAP branch is removed, the model is denoted by Bilinear Only. The GBDFNet is the full model integrating both branches. The performance metrics include ACC, Macro F1, and mAP. Results are reported as mean ± standard deviation over five fold cross-validation.

Table 5 presents the performance comparison on the tea leaf disease recognition task. Symbol * indicates that GBDFNet significantly outperforms the associated baseline model with 0.05 significant level under paired *t*-test. The corresponding CI values are also given in Table 5. The GAP Only baseline demonstrates competent performance with an mAP of 93.35%, indicating that global semantic features are effective for distinguishing distinct disease and pest-damage categories. However, the Bilinear Only variant significantly outperforms the baseline, achieving an mAP of 96.73%. This suggests that second-order statistical features are crucial for capturing subtle textural patterns that global pooling tends to smooth out.

Notably, the full GBDFNet model achieves the highest performance across all metrics, reaching an mAP of 98.04%. This improvement confirms that although bilinear features are dominant for texture-based detection, fusing them with global contextual information from the GAP branch provides complementary cues that further refine the decision boundaries, leading to superior performance against background noise.

Tea leaf-age quality grading is a fine-grained classification task requiring the discrimination of subtle visual differences between adjacent grades. In Table 6, symbol * indicates that GBDFNet significantly outperforms the associated baseline model with 0.05 significant level under paired *t*-test. The corresponding CI values are also given in Table 6. As shown in Table 6, the GAP Only model exhibits the lowest performance with ACC = 84.33%. This indicates instability in distinguishing fine-grained classes due to the loss of local texture details during global pooling. The Bilinear Only variant improves the accuracy to 85.14% and reduces variance, demonstrating the efficacy of feature correlation modeling for grading. However, the proposed GBDFNet yields the best overall performance, achieving an accuracy of 85.58% and an mAP of 95.99%. The synergy between the two branches allows the model to simultaneously leverage global maturity cues and intricate texture correlations. This results in a more discriminative feature representation for precise grade estimation.

The ablation study demonstrates that the GBDFNet significantly outperforms the GAP Independent, Bilinear Independent, GAP Only, and Bilinear Only models. Moreover, neither the GAP nor the Bilinear branch alone is sufficient to achieve optimal performance across both tasks. The GAP Only configuration struggles with fine-grained grading; whereas the Bilinear Only setup, though strong in texture analysis, lacks the global contextual grounding provided by the GAP branch. The proposed GBDFNet effectively integrates these complementary features. This validates the necessity of the dual-branch design for multi-task tea leaf analysis.

## 4. Discussion

This study proposes GBDFNet for tea leaf disease recognition and leaf-age quality grade classification. Although our experimental results demonstrate superior performance in terms of accuracy and inference speed compared to baseline models, it is crucial to delve into the underlying mechanisms driving this success, its practical implications for smart agriculture, and the current limitations that pave the way for future research.

### 4.1. Biological Interpretation

The exceptional performance of GBDFNet can be grounded in the biological characteristics of tea leaves. Tea leaf diseases often manifest as localized, visually distinct topological alterations. GAP preserves the existence of these distinct spatial activations. Conversely, tea leaf grades are determined by holistic textural uniformity and trichome distribution—features that lack distinct geometric boundaries but exhibit strong channel-wise co-occurrences. The self-bilinear branch mathematically extracts exactly this co-occurrence, acting as a texture-specialized pathway. Tea leaf grading fundamentally relies on the morphological distinction between tender buds and mature leaves. Biologically, this distinction manifests in the cellular structure: tender buds possess densely packed parenchyma cells with thinner cell walls, resulting in fine-grained, high-frequency texture patterns. In contrast, mature leaves exhibit larger, loosely arranged cells with thicker walls and more prominent veins, creating coarser, low-frequency textures.

**Figure 2:**
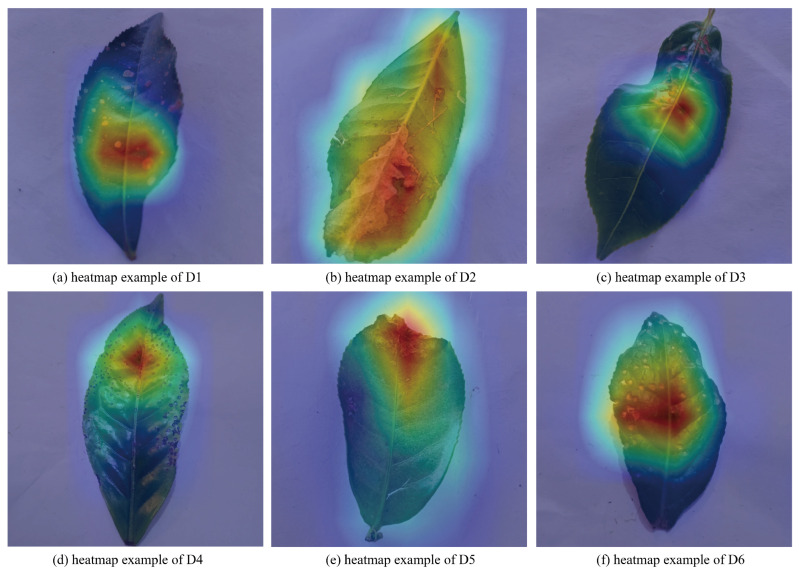
The heatmap visualization of attention regions of the proposed algorithm for tea leaf disease recognition task. Red represents high attention, whereas blue represents low attention.

To interpret the decision-making logic of GBDFNet, we employed gradient-weighted class activation mapping (GradCAM) to visualize the class-discriminative regions, as shown in Figure 2. By generating heatmaps, we can visualize which regions of the image the model focuses on during prediction, helping to understand what the model sees. This is widely applied in interpretability analysis of image classification tasks [36].

**Figure 3:**
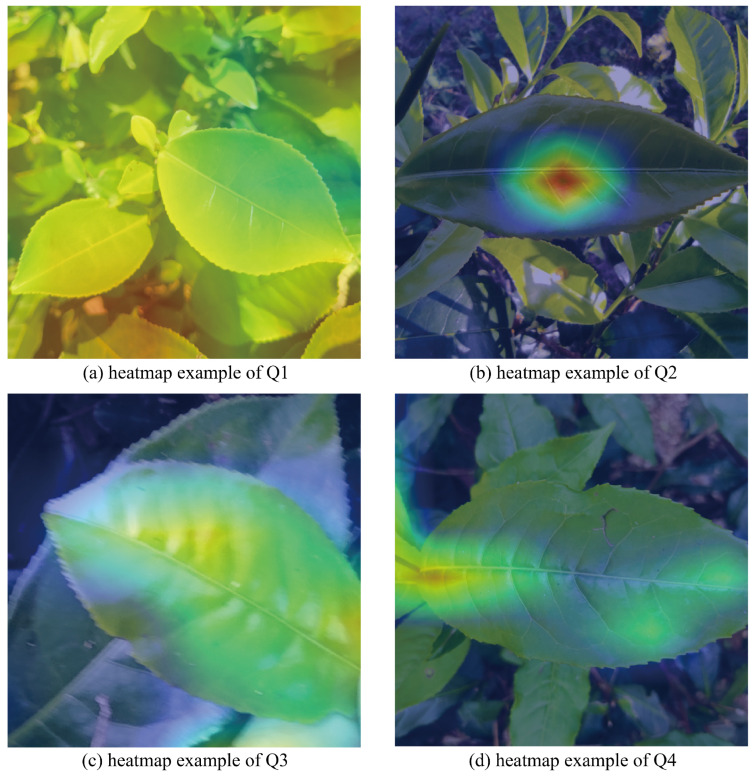
The heatmap visualization of attention regions of the proposed algorithm for tea leaf-age quality grading task. Red represents high attention, whereas blue represents low attention.

The results reveal a significant advantage of our fusion strategy. Although conventional models tend to activate over the entire leaf shape or background, GBDFNet consistently concentrates its attention on specific pathological textures and structural details. For example, in Figure 2a, the GBDFNet focuses on irregular brown lesions of tea algal leaf spot disease D1. In Figure 3a, the GBDFNet focuses on high quality tea leaf area of grade Q1.

Notably, the activation maps exhibit a sparse yet precise distribution. Instead of uniformly covering the entire diseased area, the model identifies and highlights the most diagnostic sub-regions. This behavior indicates that the self-bilinear fusion module effectively suppresses redundant background information, forcing the network to learn robust local semantics. Although some minor lesion boundaries show lower activation intensity, this focus-on-key-features mechanism enhances the model’s generalization capability, preventing it from overfitting to the specific size or shape of the lesions.

**Figure 4:**
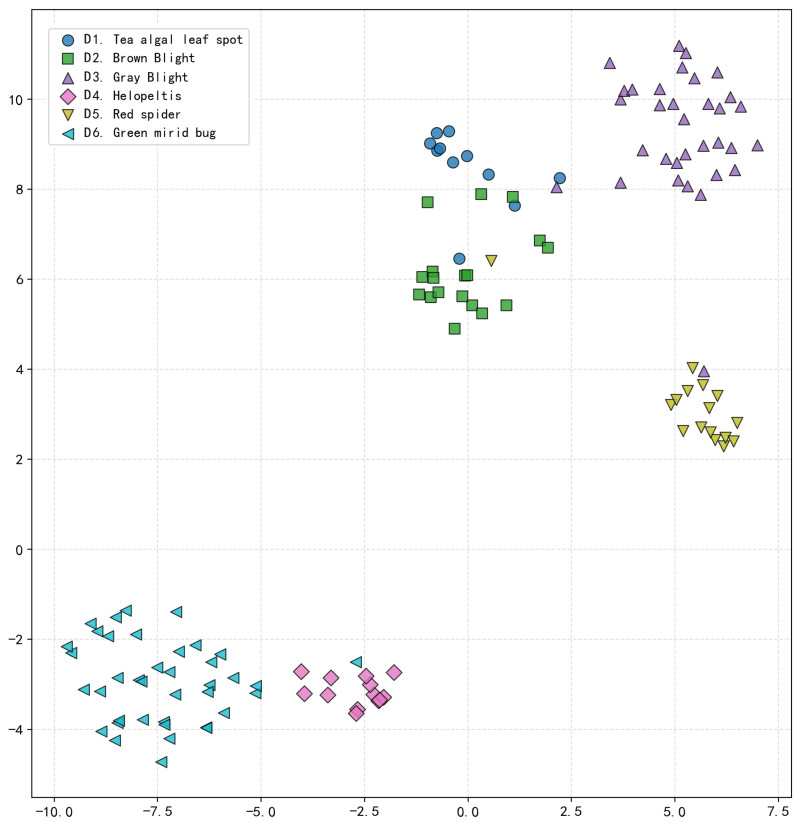
t-SNE visualization of fused feature embeddings of the proposed algorithm for tea leaf disease recognition task. Each point represents a tea leaf sample projected into a 2D space.

**Figure 5:**
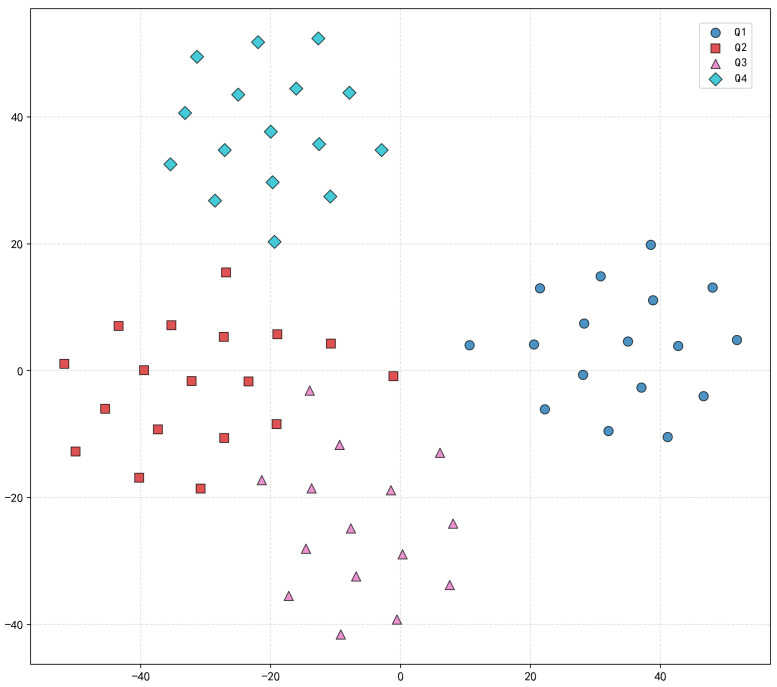
t-SNE visualization of fused feature embeddings of the proposed algorithm for tea leaf-age quality grading task. Each point represents a tea leaf sample projected into a 2D space.

To further validate the effectiveness of the learned representations, we visualized the high-dimensional fused features using t-distributed stochastic neighbor embedding (t-SNE) [37], as presented in Figure 4 and Figure 5. This is a nonlinear dimensionality reduction algorithm, commonly used for the visualization of high-dimensional data.

The embedding space exhibits two desirable properties. First, tea leaf samples belonging to the same category (e.g., D1 or Q1) cluster tightly together. This suggests that GBDFNet is robust against intra-class variations such as lighting conditions, leaf orientations, and occlusion. Second, different disease classes and health grades are well-separated with clear decision boundaries in the low-dimensional projection (e.g., D4 vs. D6 in Figure 4 or Q2 vs. Q4 in Figure 5).

It is worth observing that slight overlaps occur between certain classes (e.g., D1 vs. D2 in Figure 4). These ambiguities reflect the inherent visual similarity of certain tea leaf diseases, which poses challenges even for human experts. However, our fused features significantly reduce these overlapping regions. This confirms that combining first-order global statistics with second-order bilinear interactions enables the model to capture subtle differential cues, thereby constructing a highly structured and discriminative feature space.

The superior performance of GBDFNet is about how the model sees the leaf. We analyzed the attention maps and found that standard models often get distracted by the background or the overall leaf shape. Our dual branch network behaves differently. It forces the model to focus on the diagnostic parts. For disease and pest-damage detection the network highlights the specific lesion boundaries. For leaf-age quality grading it focuses on the fine trichomes and vein structures. This proves that our self bilinear branch successfully captures the micro textures that global pooling usually smooths out. This targeted attention mechanism is a major advantage. It makes the model robust against complex backgrounds found in real fields. It mimics the way a human expert inspects a leaf by checking specific indicators rather than just glancing at the whole image.

### 4.2. Deployment Feasibility

Precision agriculture requires models that can run on low-power devices. We leveraged MobileNetV3-Small as the backbone and restricted the bilinear expansion to a compressed 128-dimensional subspace. This design ensures low FLOPS and high inference speed on edge devices. We acknowledge the parameter count is higher than the absolute minimum, but the trade-off yields a model that is significantly faster in real-world execution.

The superior inference speed 0.29 ms of GBDFNet compared to lightweight baselines like MobileNetV3-Small with 0.69 ms can be attributed to hardware-level optimization differences. Although MobileNetV3-Small minimizes parameter count through depthwise separable convolutions, these operations often suffer from low parallelism and high memory access costs on modern GPUs. In contrast, GBDFNet leverages standard convolutions and matrix multiplications in the bilinear branch. These operations are highly optimized in deep learning libraries like PyTorch and cuDNN, allowing for better parallel processing and reduced latency despite the slightly higher parameter count. Consequently, our model achieves a better balance between model capacity and real-time execution speed.

As demonstrated in Section 3, our model achieves an inference time of approximately 0.29 ms and maintains real-time performance on embedded platforms like the Jetson Nano. This low latency is critical for real-time applications. The small parameter footprint about 5.3 M parameters enables the model to be embedded directly into handheld smart terminals used by farmers for instant quality assessment in the field. Furthermore, the computational efficiency makes GBDFNet suitable for integration into agricultural drones or autonomous harvesting robots. These systems can perform on-board, real-time grading during flight or movement. This enables precision agriculture strategies such as selective harvesting of only top-grade buds, thereby reducing labor costs and minimizing damage to the plants.

### 4.3. Limitations

Despite the promising results, we acknowledge certain limitations in the current framework. First, although GBDFNet excels in classifying single or sparsely overlapping leaves, its performance degrades in scenarios with extreme occlusion, where multiple leaves are tightly clustered or heavily overlapped. In such cases, the extracted global features may represent a mixture of different grades, leading to misclassification. Second, the current model relies on supervised learning with manually annotated datasets, which can be labor-intensive to scale across different tea varieties and growing seasons. Third, Group-wise splitting based on metadata would be the ideal way to prevent information leakage. However, this level of granular metadata was not available for the public datasets we used. We also acknowledge that the model may potentially learn dataset-specific features, as the disease and leaf-age quality images come from different sources with different backgrounds. This is a known challenge in multi-source learning.

In conclusion, the GBDFNet bridges the gap between high-accuracy deep learning and efficient edge computing, offering a biologically interpretable and practically deployable solution for modern tea production.

## 5. Materials and Methods

In this section, the data materials are introduced. The overall framework of the proposed network is shown in detail.

### 5.1. Dataset and Preprocessing

To address the dual challenges of fine-grained disease diagnosis and quality assessment in tea cultivation, this study introduces TeaLeafDiseaseQuality, a comprehensive multi-task dataset constructed by integrating and curating two SOTA public repositories: teaLeafBD dataset [38] and Tea Leaf Age Quality dataset [39]. Unlike existing benchmarks that treat disease recognition and quality grading as isolated problems, TeaLeafDiseaseQuality is explicitly designed to facilitate joint learning, capturing the intrinsic biological correlations between leaf maturity and susceptibility to specific pathogens.

#### 5.1.1. Multi-Task Dataset Construction

The foundation of TeaLeafDiseaseQuality rests on extensive fieldwork conducted across the premier tea-producing regions of Bangladesh, specifically the Sylhet and Sreemangal divisions. To facilitate multi-task learning, we integrated two public repositories: the teaLeafBD dataset and the Tea Leaf Age Quality dataset. It is important to clarify that this integration creates a unified training pool for a multi-task model, rather than a multi-label dataset where each image has two annotations. Each image in our combined dataset is assigned a single label corresponding to one of two distinct tasks: disease and pest-damage recognition or leaf-age quality grading.

**Figure 6:**
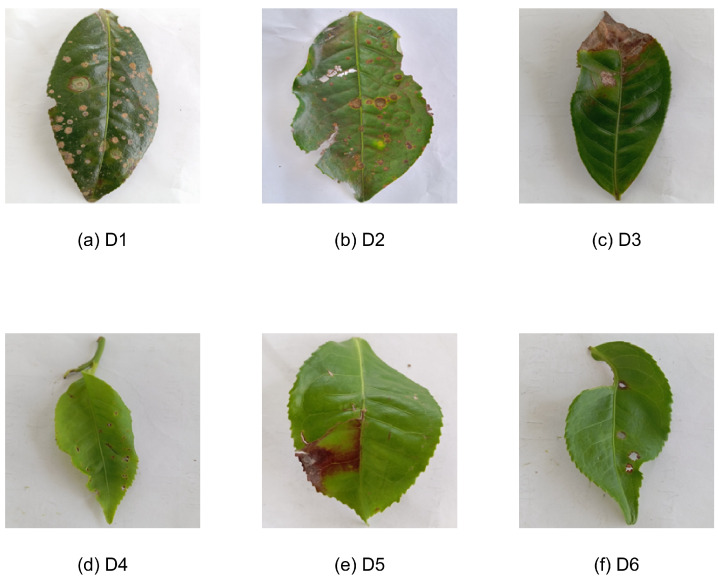
Representative samples of six distinct tea leaf diseases. The sub-images are labeled from (**a**–**f**), representing disease and pest-damage categories D1 through D6.

The disease component was compiled from eight renowned tea estates, including Malnicherra, Lakkatura, Zareen, Nurjahan, and Finlay Tea Estates [38]. Data collection was strategically timed during the monsoon season, which exacerbates the prevalence of fungal and pest infestations. A total of 5278 high-resolution images were captured using a quad-device setup to ensure device diversity and robustness against varying sensor characteristics. Figure 6 gives representative samples of tea leaf diseases. These images encompass six distinct disease and pest-damage classes representing the most economically devastating conditions in the region:

(1) Tea algal leaf spot: characterized by irregular brown lesions with gray centers, denoted by D1.
(2) Brown blight: caused by severe foliar fungal infection, manifesting as expanding necrotic spots, denoted by D2.
(3) Gray blight: presenting as grayish lesions with brown margins, denoted by D3.
(4) Helopeltis (also known as tea mosquito bug): identified by sucking damage leading to blackish necrotic patches, denoted by D4.
(5) Red spider mite: evidenced by minute reddish-brown lacerations and webbing on leaf undersides, denoted by D5.
(6) Green mirid bug: showing piercing-sucking damage resulting in deformities and discoloration, denoted by D6.

**Figure 7:**
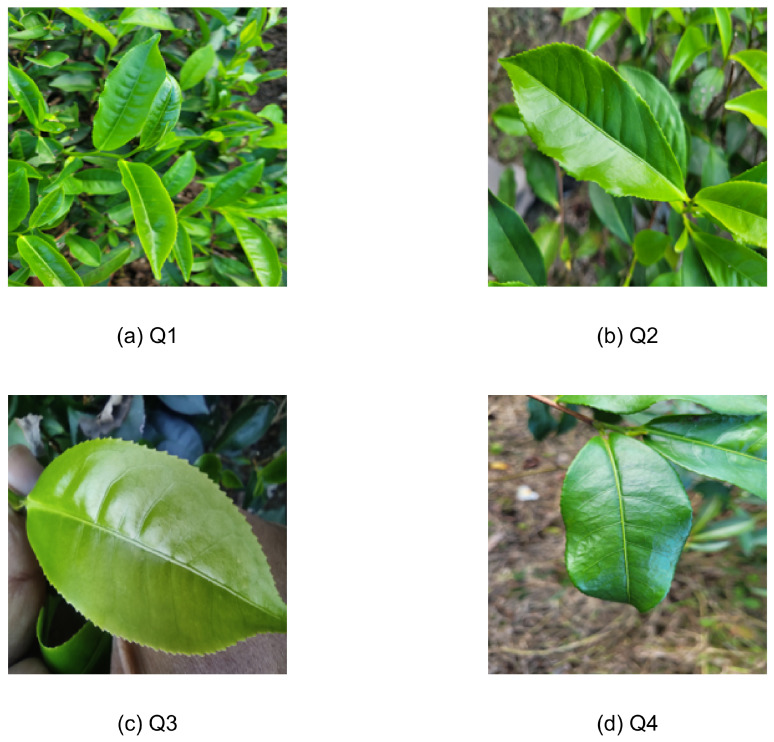
Representative samples of four distinct tea leaf qualities. The sub-images are labeled from (**a**–**d**), representing leaf-age quality grade categories Q1 through Q4.

The quality grade component was derived from the Tea Leaf Age Quality dataset, collected from the historic Malnicherra Tea Garden and surrounding areas in Moulvibazar [39]. Figure 7 gives representative samples of four tea leaf qualities. This subset consists of 2208 raw images meticulously categorized by agronomic experts into four standard commercial grades based on leaf age and plucking standards:

(1) Premium: leaves harvested within 1–2 days, representing the highest quality with superior aroma and flavor, denoted by Q1.
(2) High: leaves plucked within 3–4 days, retaining strong flavor profiles, denoted by Q2.
(3) Standard: leaves aged 5–7 days, showing moderate deterioration in quality, denoted by Q3.
(4) Low: leaves older than 7 days, characterized by significant loss of essential oils and coarse texture, generally unsuitable for premium brewing, denoted by Q4.

The Tea Leaf Age Quality dataset reported an inter-annotator reliability assessed using Cohen’s Kappa coefficient, which yielded a score of 0.836 [39], indicating “almost perfect” agreement. We relied on this high-quality annotation protocol to ensure the biological fidelity of the labels.

The final TeaLeafDiseaseQuality dataset is a union of these two sources, creating a rich resource for training a unified multi-task model. During the training process, the model learns to route features appropriately based on the image’s origin. Images from the disease dataset contribute to the optimization of the disease classification head, whereas images from the quality dataset optimize the quality grading head. This approach allows the shared backbone to learn robust features relevant to tea leaf analysis in general, whereas the task-specific heads specialize in their respective domains.

During our curation of the TeaLeafDiseaseQuality dataset, we performed a thorough manual inspection to identify and remove any obvious near-duplicates from the same sequence that could lead to data leakage. To mitigate class imbalance in the TeaLeafDiseaseQuality dataset, we conducted our experiments using a stratified five-fold cross-validation. For each class, we divided all its samples into five equal, non-overlapping parts. In each fold, one part was used for testing and the remaining four parts were used for training. This process was repeated five times, ensuring every sample was used for testing exactly once. This method guarantees that there is no overlap between the training and testing sets in any fold.

#### 5.1.2. Preprocessing and Augmentation

To enhance the robustness of the proposed GBDFNet model against the variability of field conditions and to prevent overfitting given the finite size of the dataset, a comprehensive preprocessing and augmentation pipeline was implemented.

All input images were first resized to a uniform resolution of 224 × 224 pixels, matching the input requirements of the backbone while preserving the aspect ratio through padding where necessary. Following resizing, pixel intensity values were normalized using the mean and standard deviation statistics derived from the ImageNet dataset. The mean μ = [0.485, 0.456, 0.406], and the standard deviation σ = [0.229, 0.224, 0.225]. This standardization aligns the feature distribution of our tea leaf images with the pre-trained weights of the backbone network, facilitating faster convergence and improved feature extraction.

Given the subtle visual differences between fine-grained classes (e.g., early-stage Gray Blight vs. healthy tissue), we applied a suite of stochastic data augmentation techniques exclusively to the training set. These transformations simulate diverse environmental conditions encountered in real-world deployments, such as varying camera angles, lighting changes, and occlusion. The augmentation pipeline includes:

(1) Geometric transformations: Random rotations ($\pm 30^{\circ }$), horizontal and vertical flips, and random affine transformations (scaling and shearing) to induce invariance to leaf orientation and perspective.
(2) Photometric distortions: Random adjustments to brightness (±20%), contrast (±20%), saturation, and hue to mimic variations in sunlight exposure and shadow conditions typical of outdoor tea gardens.
(3) Regularization techniques: Random erasing was applied to randomly occlude small rectangular regions of the image, forcing the model to learn distributed features rather than relying on localized cues, thereby improving generalization to partially occluded leaves.

This augmentation pipeline was specifically designed to simulate variations in field conditions and acquisition devices, thereby improving the model’s stability against environmental noise, even within the current dataset constraints.

No augmentation was applied to the validation and test sets; these subsets underwent only resizing and normalization to ensure an unbiased evaluation of the model’s performance on original data distributions. This rigorous preprocessing framework ensures that the GBDFNet model learns invariant, discriminative features capable of handling the complex visual noise of agricultural environments.

### 5.2. Proposed Architecture: Global-Bilinear Dual Fusion Network

**Figure 8:**
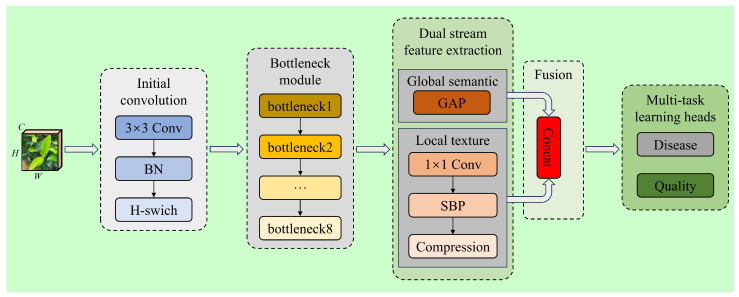
Model architecture of the proposed GBDFNet.

In this subsection, we detail the architecture of GBDFNet, an efficient yet powerful multi-task learning framework designed for the simultaneous diagnosis of tea diseases and assessment of leaf-age quality grades. As illustrated in Figure 8, the proposed method integrates a shared feature backbone with a novel dual branch feature fusion module and a multi-task prediction head. The core innovation lies in the explicit modeling of second-order texture statistics via a self-bilinear pooling branch, which complements the global semantic features extracted by standard pooling.

The backbone of GBDFNet is built based on MobileNetV3-Small [33]. The MobileNetV3-Small method has good balance between computational efficiency and representational power, making it ideal for deployment on edge devices in agricultural settings. Given an input tea leaf image $X\in R^{H\times W\times 3}$, the backbone extracts a high-level feature map $F\in R^{C\times H^{\prime }\times W^{\prime }}$, where *C* denotes the number of channels. Typically, *C* is 576 in the final layer of MobileNetV3-Small and $H^{\prime },W^{\prime }$ are the spatial dimensions.

#### 5.2.1. Initial Convolution

Given an input tea leaf image $X\in R^{H\times W\times 3}$. The initial convolution operation performs fundamental feature extraction from input image *X*. It utilizes a 3×3 convolutional (3×3 Conv) kernel with stride 2 to process the input. This operation expands the channel dimension from 3 to 16 while reducing the spatial dimensions by half. The layer incorporates batch normalization (BN) followed by h-swish activation (H-swish) function. This design efficiently captures basic visual patterns while optimizing computational resources for mobile deployment. The output feature map dimensions are H/2×W/2×16 after the initial convolution operation.

#### 5.2.2. Bottleneck Module

The bottleneck modules form the core feature extraction mechanism of the GBDFNet. It is comprised of 8 bottlenecks. The 8 bottlenecks strategically combine 3×3 and 5×5 kernels with varying strides to downsample spatial dimensions and expand channel dimensions. The configuration of the 8 bottlenecks is shown in Table 7.

**Figure 9:**
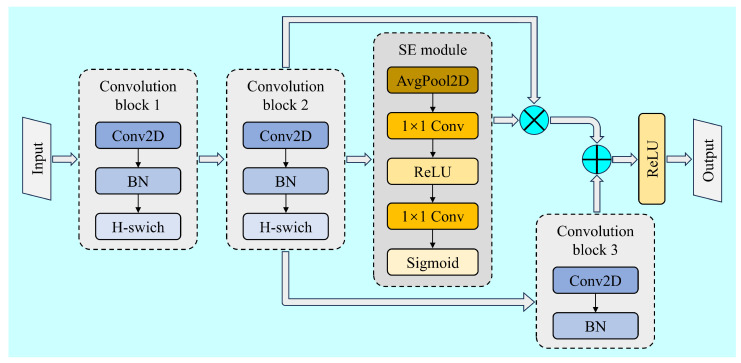
The bottleneck block used in the GBDFNet.

The bottleneck block is shown in Figure 9. This block utilizes H-swish as the non-linear activation function and incorporates squeeze-and-excitation (SE) modules where specified to enhance feature representation. Note that the block in Figure 9 consists all operations of the bottleneck module. Some operation may not be used. For example, bottleneck1 does not use SE module as given in Table 7.

#### 5.2.3. Dual-Stream Feature Extraction

Unlike traditional single-stream architectures that rely solely on global average pooling (GAP), GBDFNet splits the feature processing into two parallel streams:

(1) Global semantic stream: Captures first-order statistical information like global shape and color distribution.
(2) Local texture stream: Explicitly models second-order statistical information like channel correlations and fine-grained textures using self-bilinear pooling.

The features from both streams are fused and subsequently fed into two independent fully connected heads to predict: (1) Disease and pest-damage category and (2) Quality grade.

**Figure 10:**
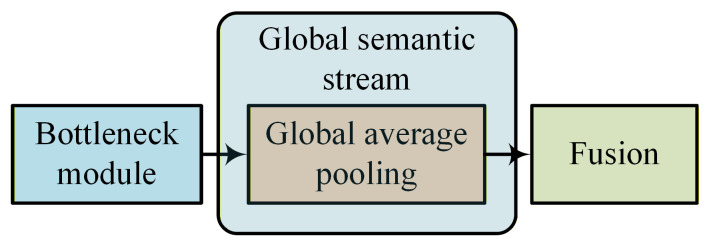
Architecture of the global semantic stream.

The first stream is the global semantic stream as shown in Figure 10. It employs standard GAP to aggregate spatial information, generating a compact global descriptor. For the feature map F, the global feature vector $v_{gap}\in R^{C}$ is computed as:

(1) $$v_{gap}\left[c\right]=\frac{1}{H^{\prime }W^{\prime }}\sum_{i=1}^{H^{\prime }}\sum_{j=1}^{W^{\prime }}F\left[c,i,j\right],$$

where *c* indexes the channel. This operation effectively captures the overall presence of disease symptoms or leaf structures but tends to smooth out subtle textural details critical for fine-grained differentiation.

**Figure 11:**
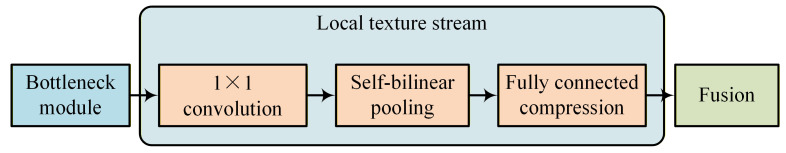
Architecture of the local texture stream.

The second stream is the local texture stream as shown in Figure 11. To address the limitation of GAP in capturing fine-grained textures (e.g., early fungal spots or leaf vein density), we introduce a self-bilinear pooling module. To mitigate the computational burden of self-bilinear pooling module, the feature maps from the backbone are first projected into a lower-dimensional subspace via a 1×1 convolutional layer. Specifically, the original 576 feature tensor is reduced to 128, yielding a compact representation $X\in R^{B\times 128\times H\times W}$ that retains spatial structure while significantly decreasing the dimensionality of the subsequent outer product. Then, the self-bilinear pooling module computes the outer product of the feature map with itself, thereby encoding pairwise interactions between all feature channels. This results in a rich representation of second-order statistics.

Formally, let $f_{i,j}\in R^{C}$ be the feature vector at spatial location (i,j). The bilinear feature matrix $Z_{i,j}\in R^{C\times C}$ is calculated as:

(2) $$Z_{i,j}=f_{i,j}f_{i,j}^{⊤}.$$

Aggregating over all spatial locations yields the global bilinear feature matrix $Z\in R^{C\times C}$:

(3) $$Z=\sum_{i=1}^{H^{\prime }}\sum_{j=1}^{W^{\prime }}f_{i,j}f_{i,j}^{⊤}.$$

Directly flattening Z would result in a very high-dimensional vector, leading to excessive memory consumption. To mitigate this, we employ a dimensionality reduction strategy inspired by compact bilinear pooling. We first apply a signed square root normalization to every element of the bilinear matrix. This operation suppresses large activation values and enhances robustness against outliers:

(4) $$\hat{Z}=\mathrm{sign}\left(Z\right)\sqrt{\left|Z\right|}.$$

The normalization is performed element-wise, meaning each entry of Z is transformed independently based on its own sign and magnitude. This step prevents a small number of dominant channels from overwhelming the subsequent projection. Subsequently, $\hat{Z}$ is flattened and passed through a projection layer to compress the feature dimension from $C^{2}$ to a compact embedding size D=256. The projection layer is a fully connected layer followed by batch normalization and ReLU. The resulting texture feature vector is denoted as $v_{bil}\in R^{D}$. Finally, L2 normalization is applied directly to $v_{bil}$:

(5) $$v_{bil}\leftarrow \frac{v_{bil}}{∥v_{bil}{∥}_{2}}.$$

This process ensures that the model explicitly learns the correlations between different filter responses, which correspond to specific textural patterns indicative of disease and pest-damage types and leaf maturity.

#### 5.2.4. Feature Fusion

To leverage the complementary nature of global semantics and local textures, we concatenate the two feature vectors:

(6) $$v_{fused}=\left[v_{gap};v_{bil}\right],$$

where [·;·] denotes concatenation. The fused vector $v_{fused}\in R^{C+D}$ serves as the comprehensive representation for downstream tasks. In our architecture, this fused vector is further processed by a bottleneck layer to reduce redundancy before branching into task-specific heads as follows.

#### 5.2.5. Multi-Task Learning Heads

The unified feature representation $v_{fused}$ is fed into two parallel classification heads, each optimized for a specific agronomic task:

(1) Disease category head: A multi-class classifier predicting the specific tea leaf disease category $y_{disease}\in \left\{0,\ldots ,K_{d}-1\right\}$, where $K_{d}=6$ (diseases D1 to D6).
(2) Quality grade head: A multi-class classifier predicting the tea leaf-age quality grade $y_{grade}\in \left\{0,\ldots ,K_{g}-1\right\}$, where $K_{g}=4$ (grades Q1 to Q4).

Each head consists of a fully connected (FC) layer followed by a Softmax activation function.

#### 5.2.6. Loss Function and Optimization

To train the network end-to-end, we define a composite multi-task loss function $L_{total}$ that aggregates the cross-entropy (CE) losses from all two tasks:

(7) $$L_{total}=\lambda_{1}L_{disease}+\lambda_{2}L_{grade},$$

where $L_{task}=-\sum_{k}y_{k}\log\left(p_{k}\right)$ is the standard CE loss for each respective task. The hyperparameters $\lambda_{1},\lambda_{2}$ control the relative importance of each task. In our experiments, we set $\lambda_{1}=1.0$ and $\lambda_{2}=1.0$. We assign equal weights to tea disease and pest-damage recognition and leaf-age quality grading classification, indicating that both tasks are of equal importance. Users can adjust these weights to prioritize one task over the other according to their specific needs.

The model parameters are optimized using the AdamW optimizer with an initial learning rate of $1\times 10^{-3}$, coupled with a cosine annealing scheduler to dynamically adjust the learning rate during training. This optimization strategy ensures stable convergence while allowing the model to escape local minima, ultimately yielding a robust system capable of precise, multi-faceted tea leaf analysis.

## 6. Conclusions

In this paper, we proposed a global-bilinear dual fusion network to resolve the inherent feature conflicts in multi-task tea leaf assessment. By explicitly decoupling first-order spatial features and second-order covariance features, our architecture mimics the holistic diagnostic approach of human experts. Experimental results confirm that this dual-routing method significantly improves multi-task accuracy without compromising computational efficiency. Current evaluation demonstrates the GBDFNet model’s robustness in handling intra-class variations rather than broad domain generalization.

Future work will address these challenges through the following directions. Knowledge distillation or tensor decomposition may be used to compress the bilinear layers, making the model more hardware-friendly without sacrificing accuracy. We plan to integrate object detection heads or attention mechanisms that can localize and separate individual leaves before classification [40], or adopt instance segmentation techniques to handle complex clusters. Moreover, to enhance performance across varying environmental conditions and tea cultivars, we will explore unsupervised domain adaptation and few-shot learning strategies. Finally, incorporating spectral information alongside RGB images could provide complementary chemical composition data [41], we will further improve the accuracy of grade estimation beyond visual texture alone.

## Figures and Tables

**Table 1:** Hyperparameters used in all experiments.

Hyperparameter	Value
Epochs	50
Batch size	32
Learning rate	$1\times 10^{-3}$
Optimizer	AdamW
Weight decay	$1\times 10^{-5}$

**Table 2:** Comparative analysis of computational complexity and inference efficiency. **Bold** indicates the best performance. GBDFNet achieves the optimal balance between accuracy and efficiency.

Model	Params (M)	FLOPS (M)	Time (ms)
MobileNetV3-Small	**0.934**	**60.87**	0.69
ShuffleNet	1.266	151.75	0.62
MnasNet	3.118	335.042	0.73
YOLOv11n-cls	1.204	182.79	0.59
GBDFNet (Ours)	5.306	68.71	**0.29**

Note: Params and FLOPS are calculated based on an input size of 224×224. Inference time is measured on an NVIDIA RTX 3090 GPU.

**Table 3:** Performance comparison of different models on the tea leaf disease task. Results are reported as mean ± standard deviation. The best results are highlighted in **bold**. N/A means not available.

Model	ACC (%)	F1 (%)	mAP (%)	CI
MobileNetV3-Small	86.40±3.01 *	86.58±1.82	94.57±1.70	[−8.20, −5.23]
ShuffleNet	45.59±7.78 *	40.36±9.41	80.64±2.38	[−50.80, −44.24]
MnasNet	71.75±4.21 *	67.56±6.12	88.23±1.54	[−23.66, −19.07]
YOLOv11n-cls	64.63±6.12 *	64.34±5.02	82.74±6.83	[−30.53, −26.44]
GBDFNet (Ours)	93.12±2.43	92.51±2.86	98.04±1.20	N/A

Note: * means that the GBDFNet shows significant better performance than the associated baseline model.

**Table 4:** Performance comparison of different models on the tea leaf-age quality grading task. Results are reported as mean ± standard deviation. The best results are highlighted in **bold**. N/A means not available.

Model	ACC (%)	F1 (%)	mAP (%)	CI
MobileNetV3-Small	80.25±13.69 *	81.03±14.62	93.02±8.79	[−7.30, −4.57]
ShuffleNet	82.59±10.79 *	82.57±11.54	93.88±5.12	[−56.22, −48.07]
MnasNet	66.17±8.11 *	65.18±8.21	88.69±7.20	[−27.86, −22.03]
YOLOv11n-cls	72.35±11.63 *	72.65±13.46	87.06±7.52	[−29.79, −26.55]
GBDFNet (Ours)	85.58±11.49	86.66±11.76	95.99±8.03	N/A

Note: * means that the GBDFNet shows significant better performance than the associated baseline model.

**Table 5:** Ablation study results on tea leaf disease recognition. We compare the performance of different feature branches. The best results are highlighted in **bold**. N/A means not available.

Model	ACC (%)	F1 (%)	mAP (%)	CI
GAP Independent	85.18±2.40 *	75.01±3.24	76.92±3.39	[−8.59, −7.66]
Bilinear Independent	88.58±0.42 *	84.29±0.46	88.90±2.25	[−5.64, −3.81]
GAP Only	87.43±2.83 *	84.25±2.58	93.35±2.07	[−6.50, −5.25]
Bilinear Only	92.07±1.28 *	92.04±2.27	96.73±2.56	[−2.35, −0.11]
GBDFNet (Ours)	93.12±2.43	92.51±2.86	98.04±1.20	N/A

Note: * means that the GBDFNet shows significant better performance than the associated baseline model.

**Table 6:** Ablation study on tea leaf-age quality grading. We compare the performance of different feature branches. The best results are highlighted in **bold**. N/A means not available.

Model	ACC (%)	F1 (%)	mAP (%)	CI
GAP Independent	74.76±2.16 *	79.25±1.32	90.02±1.12	[−16.44, −9.21]
Bilinear Independent	81.45±1.47 *	81.21±3.36	83.48±1.63	[−9.43, −2.85]
GAP Only	84.33±10.41 *	83.28±13.00	91.65±5.20	[−4.37, −2.13]
Bilinear Only	85.14±7.34 *	81.64±8.26	94.32±4.53	[−4.24, −0.25]
GBDFNet (Ours)	85.58±11.49	86.66±11.76	95.99±8.03	N/A

Note: * means that the GBDFNet shows significant better performance than the associated baseline model.

**Table 7:** Configuration of the 8 bottlenecks in the GBDFNet.

Layer	Input	Output	Kernel	Stride	SE
bottleneck1	16	32	3×3	2	False
bottleneck2	32	64	3×3	2	True
bottleneck3	64	128	5×5	2	True
bottleneck4	128	128	5×5	1	True
bottleneck5	128	240	5×5	2	True
bottleneck6	240	240	5×5	1	True
bottleneck7	240	480	5×5	1	True
bottleneck8	480	576	5×5	1	True

## Data Availability

The tea leaf disease recognition dataset analyzed in this study is available from https://data.mendeley.com/datasets/744vznw5k2/3 (accessed on 11 February 2026), and the tea leaf grading dataset is available from https://data.mendeley.com/datasets/7t964jmmy3/1 (accessed on 11 February 2026).

## Abbreviations

The following abbreviations are used in this manuscript:

DL	Deep learning
CV	Computer vision
CNNs	Convolutional neural network
SOTA	State-of-the-art
GAP	Global average pooling
ACC	Accuracy
mAP	mean average precision
F1	F1-score
FLOPS	Floating-point operations per seconds
